# Epidemiology and risk factors for pyogenic liver abscess in the Calgary Health Zone revisited: a population-based study

**DOI:** 10.1186/s12879-021-06649-9

**Published:** 2021-09-10

**Authors:** Jennifer A. Losie, John C. Lam, Daniel B. Gregson, Michael D. Parkins

**Affiliations:** 1 Department of Medicine, University of British Columbia, Vancouver, Canada; 2 Department of Medicine, University of Calgary, Calgary, Canada; 3 Department of Microbiology and Infectious Diseases, Calgary Zone Alberta Health Services, University of Calgary, 3330 Hospital Drive NW, Calgary, AB T2N 4N1 Canada; 4 Alberta Provincial Laboratories, Calgary, Canada; 5 Department of Pathology and Laboratory Medicine, University of Calgary, Calgary, Canada

**Keywords:** Biliary disease, Antibiotic resistance, *Streptococcus anginosus* group, *Klebsiella pneumoniae*, Bacteremia, Polymicrobial

## Abstract

**Background:**

Pyogenic liver abscess (PLA), although uncommon in North America, is associated with significant morbidity and mortality. We sought to re-examine the epidemiology, risk factors, and outcomes of PLA in a large, diverse Canadian health zone.

**Methods:**

All Calgary Health Zone (CHZ) residents aged ≥20 with PLA between 2015 and 2017 were identified. Incidence and mortality rates were calculated using census data. Risk factors for PLA were identified using a multivariate analysis. Data was compared to 1999–2003 data, also collected in the CHZ.

**Results:**

There were 136 patients diagnosed with PLA between 2015 and 2017. Incidence rate during this period increased significantly relative to 1999–2003 (3.7 vs 2.3 cases/100,000 population, p < 0.01), however, mortality rates remained similar. The microbiological composition of PLA did not change over this 15-year time period but the number of antimicrobial resistant isolates did increase (8% vs 1%, p = 0.04). The greatest risk factors for PLA relative to general populations included current malignancy, liver-transplant, end-stage renal disease, and cirrhosis. Thirty-day mortality was 7.4% and independent risk factors included polymicrobial bacteremia, absence of abscess drainage, congestive-heart failure, a history of liver disease, and admission bilirubin.

**Conclusions:**

Pyogenic liver abscess is a health concern with rising incidence rate. The increasing prevalence of comorbidities in our population and factors that are associated with risk of PLA suggests this will continue to be an emerging diagnosis of concern. Increasing prevalence of antibiotic resistant organisms compounding unclear optimal treatment regimens is an issue that requires urgent study.

## Background

Pyogenic liver abscess (PLA) is a significant health concern, with highest incident rates reported in Asia. Endemic in Taiwan, PLA has been steadily increasing in incidence, most recently 15.45/100,000 population in 2011 [1]. Although less frequent in North America, PLA still has an estimated incidence between 2.3 and 3.6/100,000 population in Canada and the United States respectively. Furthermore, this condition is associated with significant morbidity and has a mortality risk between 6 and 10% [2, 3].

The pathogenesis of PLA is varied. These abscesses can develop from; (i) biliary tree pathology (e.g. ascending cholangitis), (ii) ascending gastrointestinal tract infections via the hepatic portal vein, (iii) systemic bacteremia via the hepatic artery, (iv) contiguous spread, (v) direct inoculation from trauma or an invasive procedure, and (vi) cryptogenically [4]. Biliary pathology represents the most common etiology ranging from 11 to 37% of causes in Southeast Asia [5]. Approximately 75% of hepatic abscesses involve the right lobe, 20% in the left lobe, and 5% in the caudate lobe [6].

The microbiology of PLA varies greatly—with geography recognized as an important factor. Worldwide, *Escherichia coli* is the most common culprit [1]. Within a Canadian population the *Streptococcus anginosus* group (44%), *Klebsiella* species (27%), anaerobes (20%), and *Escherichia coli* (16%) were cultured most frequently [3]. Similar to Canada, *Streptococcus* and *Enterococcus* species were the most prevalent in the United States (29.5%), followed by *Escherichia* (18.1%), *Klebsiella* species (9.2%), and anaerobes (8.6%) [2]. However, multiple studies from Taiwan and other parts of Asia have found *Klebsiella* species to be most common (approximately 68–79% of cultured organisms) with increasing concern about the prevalence of extended spectrum beta-lactamase (ESBL) producing species as well as hypervirulent mucoviscous strains [7, 8].

In this study, we sought to compare the epidemiology and risk factors for PLA in the Calgary Health Zone (CHZ) between 2015 and 2017 to data reported in a similar study by Kaplan et al. captured between 1999 and 2003.

## Methods

### Study population

The CHZ provides all laboratory and inpatient medical care to a population of approximately 1.3 million residents [9]. All patients aged ≥20 years within the CHZ diagnosed with a PLA between 2015 and 2017 were included. Comparator population numbers and rates of comorbidities of CHZ residents were determined using annual census data [10]. The CHZ is composed of approximately 64% Caucasian, 22% Asian, 5% Indigenous, and 9% other ethnicities [11].

### Data sources

Study subjects were identified using the International Classification of Diseases 9 and 10 (ICD-9 & ICD-10) codes for hospitalized individuals diagnosed with PLA and cross referenced to the Calgary Laboratory Services (CLS) database to identify all microbiological specimens taken from liver aspirations. CLS is a regional service that accepts and processes all microbiological samples within CHZ. Once individuals were identified using these two methods, chart reviews (using Sunrise Clinical Manager electronic medical record) were conducted using a standardized form to record demographics, comorbidities, hospitalization length, mortality, occurrence of relapse, as well as microbiological, radiographic, and biochemical data. This study received approval through the Conjoint Health Research Ethics Board at the University of Calgary (REB18-2013).

We compared the results from our data set (collected between 2015 and 2017) to the data published by Kaplan et al. (collected between 1999 and 2003) [3] in order to examine the changes in epidemiology, etiology, and risk factors for PLA over 15-years. Both our study and the study by Kaplan et al. are retrospective population-based studies from the Calgary Health Zone and results were reported on a per capita basis.

### Definitions

Definitions were based upon those used in the Kaplan et al. study for consistency in comparison. PLA was diagnosed if at least one of the following conditions was met: (1) positive microbiological culture from aspirated liver abscess; (2) identification of systemic bacteremia in conjunction with radiographic findings consistent with liver abscess; or (3) improvement of radiographic lesions consistent with liver abscess with antimicrobial therapy and with appropriate exclusion of alternate diagnoses.

Mortality was calculated as 30-day, in-hospital mortality which was consistent with criteria from Kaplan et al. to allow for comparison. Antimicrobial resistant organisms were defined as either: (1) methicillin-resistant *Staphylococcus aureus* (MRSA), (2) vancomycin-resistant *Enterococcus* (VRE) species, (3) penicillin-resistant *Streptococcus pneumoniae*, or (4) any Gram-negative organism resistant to one or more of: a fluoroquinolone, aminoglycoside, 3rd generation cephalosporin, or carbapenem.

### Statistical analysis

Non-normally distributed variables were reported as medians with interquartile ranges (IQR) and compared using the rank-sum test for pairs or median test for multiples. Differences in proportions among categoric data were assessed using Fisher’s exact test for pair-wise comparisons and the chi-square test for multiple groups and cohorts. The incidence rate of PLA was calculated by dividing the incident CHZ cases by the defined regional population at risk. Risk factors for PLA were quantified by dividing the occurrence incidence rate among those with a given factor by those without. Extracted demographic data were used to determine the population at risk for assessment of age and gender [10]. For other potential risk factors, the population at risk was ascertained or estimated using local patient registry data [10, 12, 13]. Risks are expressed as incidence rate ratios (RR) and reported with 95% confidence intervals (CI). Mortality rates were calculated using all-cause 30-day deaths. A logistic regression model was developed to assess factors independently associated with 30-day mortality. Factors associated with death in univariate analyses (p < 0.1) were included and backward stepwise elimination was performed to develop the final model. Model calibration was assessed using the Hosmer–Lemeshow goodness of fit test and discrimination was assessed using the area under the receiver operator characteristic curve. Model results are reported as odds ratios (OR) with 95% CI. A p-value < 0.05 was considered statistically significant for all comparisons. All statistical calculations were performed using STATA 16.1 (College Stn., TX).

## Results

Between January 1, 2015 and December 31, 2017, 136 patients were admitted to one of the four hospitals in the CHZ for PLA. Recurrent episodes in the same subject were not separately analyzed.

### Incidence rate

Mean annual incidence rate per annum based on population from 2015 to 2017 was 3.7 cases/100,000 population. Compared to the 1999–2003 cohort, incidence rate has increased (2.3 cases/100,000 population between 1999 and 2003, p < 0.01).

**Fig. 1:**
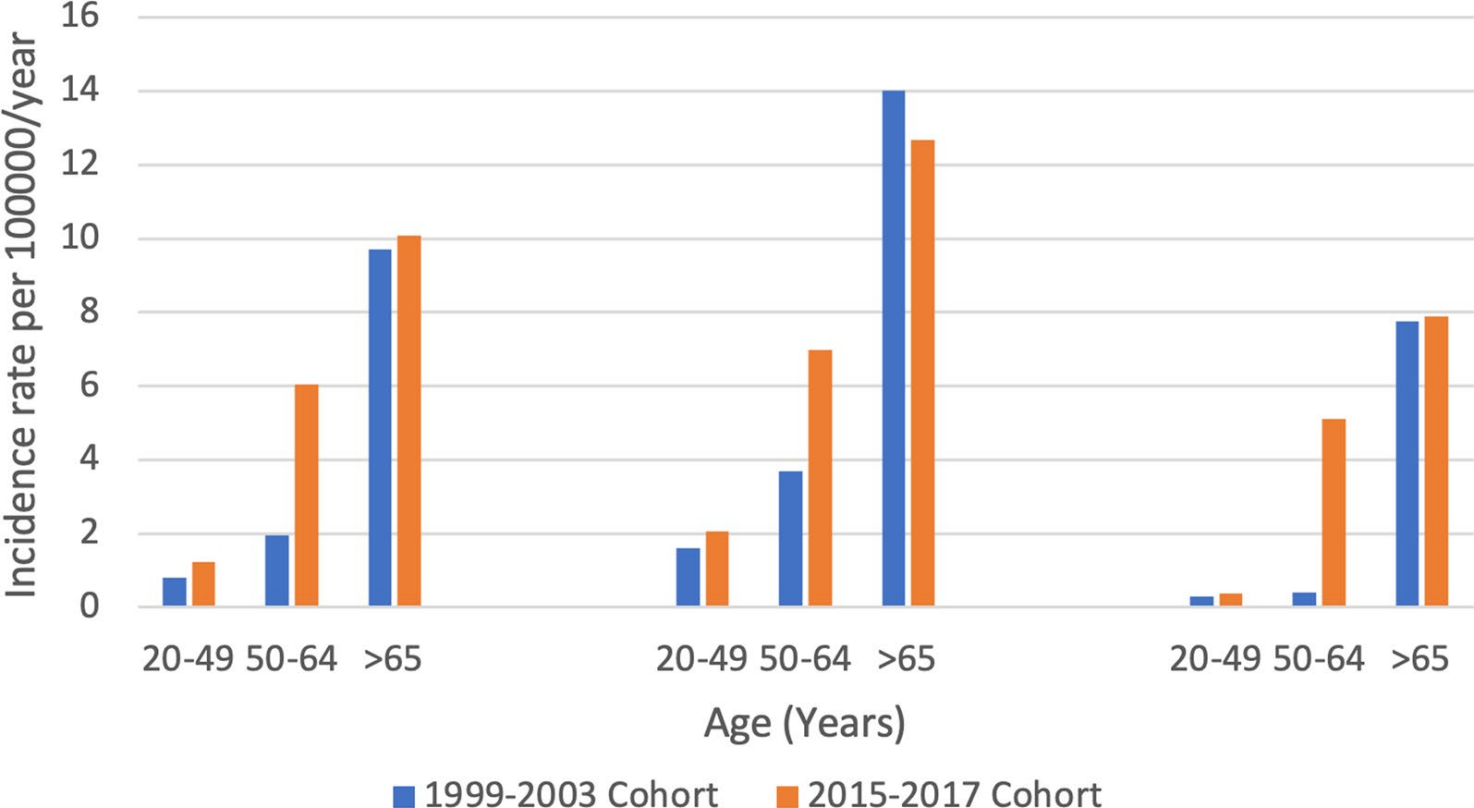
Incidence rate of PLA as a function of patient demographics over time

Similar to 15-years ago, incidence rate of PLA was higher among males compared to females (4.7 vs 2.7/100,000 population between 2015 and 2017, RR = 1.74 (1.08–2.79 95% CI), p = 0.01). Additionally, incidence rate of PLA increased with age. Compared to 15 years ago, the highest increase in incidence rate was observed in the 50–64 age group and predominantly in females (Fig. 1).

### Diagnosis

In our cohort, PLA was diagnosed from aspiration of a hepatic abscess in 89 patients (65%), blood culture with consistent imaging findings in 34 patients (25%), and improved imaging consistent with PLA in response to therapy in the context of negative cultures in 13 (10%). The number diagnosed via these mechanisms were not significantly different compared to 15 years ago. Although the overall proportion of patients diagnosed via liver aspiration was not different, the proportion of patients who underwent liver aspiration was lower (74% vs 87%, p = 0.02) but the proportion of positive cultures was higher in our cohort compared to 15 years ago (88% vs 71%, p = 0.006).

Similar to Kaplan et al. computed tomography (CT) detected PLA in 98% of cases (99% Kaplan et al.) and ultrasound detected PLA in 93% of cases (91% Kaplan et al.). In our study, a smaller proportion of patients had a single PLA (2015–2017: 45% vs 1999–2003: 61%, p = 0.023), more patients had 2 abscesses (2015–2017: 20% vs 1999–2003: 10%, p = 0.047), and a similar proportion of patients had ≥ 3 abscesses (2015–2017: 35% vs 1999–2003: 29%, p = 0.25).

PLA was confined to the right lobe in 83 of our cohort (61%) which was numerically lower than previously (72%) but this was not statistically significant (p = 0.081). Isolated left lobe abscesses were identified in 16 patients (12%), similar to 15% in the prior data. Our cohort had a higher number of patients with PLA involving both lobes, 37 patients (27%), compared to 13% in the Kaplan et al. study (p = 0.011). Pylephlebitis was observed in 10% of PLA in our cohort and was diagnosed by either CT scan or ultrasound. Pylephlebitis was associated with current malignancy (p = 0.03) and not other comorbidities recorded in our study. Patients with this complication were not more likely to die.

Confirmed or suspected PLA was the admitting diagnosis in 62 of our subjects (46%) which is higher compared to 15 years ago when only 27% of patients were known to have PLA on admission (p = 0.006).

### Source of PLA

Forty-six patients in our cohort (34%) had a biliary cause for their PLA identified. There were 40 patients (29%) with no apparent source and PLA was determined to be cryptogenic. Other causes of PLA are outlined in Table 1. Those who had cryptogenic PLA were more likely to have monomicrobial abscesses (p = 0.035) and were more likely to have infections with *Klebsiella* species (p = 0.01) whereas patients with *E. coli* or Enterococcal species as the culprit organisms were less likely to have cryptogenic PLA (p = 0.034, p = 0.012).

**Table 1:** Comparing the cause and associated-factors of PLA over time

Etiology	Prevalence between 1999 and 2003	Prevalence between 2015 and 2017	p-value
Cryptogenic	56% (40/71)	29% (40/136)	**< 0.001**
Biliary	24% (17/71)	34% (46/136)	0.09
Choledocholithiasis	7% (5/71)	7% (10/136)	0.59
Cholecystitis	7% (5/71)	15% (20/136)	0.08
Ascending cholangitis	6% (4/71)	11% (15/136)	0.15
Common bile duct stricture	4% (3/71)	4% (5/136)	0.56
Pylephlebitis	1% (1/71)	10% (14/136)	**0.01**
Cholangiocarcinoma	1% (1/71)	4% (6/136)	0.24
Bile leak post-cholecystectomy	1% (1/71)	1% (2/136)	0.73
Choledochojejunostomy	1% (1/71)	0% (0/136)	0.34
Nonbiliary	20% (14/71)	36% (49/136)	**0.01**
Neutropenia	4% (3/71)	1% (1/136)	0.12
Perforation	3% (2/71)	4% (5/136)	0.55
Pneumonia/empyema	3% (2/71)	0% (0/136)	0.12
Diverticulitis	1% (1/71)	12% (16/136)	**0.006**
Perinephric abscess	1% (1/71)	0% (0/136)	0.34
Endocarditis	1% (1/71)	0% (0/136)	0.34
Dental abscess	1% (1/71)	1% (1/136)	0.57
Appendicitis	1% (1/71)	1% (2/136)	0.73
Traumatic liver injury	1% (1/71)	1% (1/136)	0.57
Gastroenteritis/colitis	0% (0/71)	5% (7/136)	**0.05**
Post-surgical complication	0% (0/71)	3% (4/136)	0.18
Superinfected liver metastasis	0% (0/71)	2% (3/136)	0.28
Pancreatitis	0% (0/71)	1% (2/136)	0.43
Other^a^	0% (0/71)	1% (2/136)	0.43

^a^Occlusion of colonic sinus tract, pharyngitis

### Risk factors

In our cohort, 63% of patients were male, compared to 72% of patients in the Kaplan et al. cohort (p = 0.14). The mean age was 60.7 years ± 16 in our cohort compared to 62.4 ± 14 years in the Kaplan et al. group. Prevalent co-morbidities associated with increased risk of PLA between 1999 and 2003 included: liver transplant, diabetes mellitus, alcohol use disorder, and history of malignancy. Prevalent co-morbidities with PLA in our data set included: diabetes (24%), current malignancy (19%), ischemic heart disease (14%), chronic obstructive pulmonary disease (7.4%), cirrhosis (4.4%), history of congestive heart failure (3.7%), end stage renal disease (3%), and liver transplant (3%), each of which was significantly more common (p < 0.05) than in the general population (Table 2). Seventy five individuals (55%) had one of the above listed comorbidities, while 61 individuals (45%) did not. Rates of other comorbidities including rheumatoid arthritis and heavy alcohol use did not differ from general populations (p > 0.05).

**Table 2:** Population-based factors associated with increased risk of PLA

Underlying condition	Number of patients (n = 136)	Estimated number with underlying condition at risk^a^	Incidence Rate per 100,000^b^	RR (95% CI)	p value
Diabetes	33	281,890	11.7	3.81 (2.58–5.65)	< 0.0001
Current malignancy	26	18,931	137.3	45.13 (29.44–69.18)	< 0.0001
Ischemic heart disease	19	135,675	14.00	4.19 (2.58–6.81)	< 0.0001
Chronic obstructive pulmonary disease	10	84,285	11.86	3.35 (1.76–6.37)	0.001
Cirrhosis	6	8,975	66.85	18.65 (8.23–42.27)	< 0.0001
Congestive heart failure	5	53,851	9.28	2.54 (1.04–6.21)	0.05
End stage renal disease	4	3,999	100.02	27.51 (10.18–74.36)	< 0.0001
Liver transplant	4	2,643	151.34	41.63 (15.40–112.47)	< 0.0001

^a^Estimated 3 year prevalence of the underlying condition in the CHZ

^b^Incidence rate of PLA among those with the underlying condition

### Microbiology

Forty-four (32%) of subjects had polymicrobial PLA. Similar to the Kaplan et al. study, the most commonly identified organism was *Streptococcus anginosus* group (previously known as *Streptococcus milleri* group). One *Streptococcus anginosus* isolate had intermediate sensitivity to penicillin, none were resistant to cephalosporins. *Klebsiella* species remain the second most commonly isolated organism (Table 3). Six infections (4.4%) were nosocomial. Eleven patients had resistant organisms identified; 4 were extended-spectrum beta-lactamase producing organisms, one an AmpC-producing *Klebsiella*, one MRSA, one VRE, and the remainder were resistant Gram-negatives. There were no carbapenem-resistant organisms. The number of resistant organisms was significantly higher in our cohort compared to the Kaplan et al. cohort (p = 0.04).

**Table 3:** Microbial constituents in PLA between cohorts

Factor	1999–2003 Cohort	2015–2017 Cohort	p value
Polymicrobial	35/71 (49%)	44/136 (32%)	**0.01**
Nosocomial PLA	6/71 (9%)	6/136 (4.4%)	0.19
*Streptococcus anginosus* group	31/71	54/136	0.34
*Klebsiella *species	19/71	34/136	0.45
Anaerobes^a^	14/71	22/136	0.32
*Escherichia coli*	11/71	24/136	0.43
*Enterococcus* species	5/71	12/136	0.44
*Staphylococcus aureus*	4/71	2/136	0.11
Other bacteria	11/71	26/136	0.33
Culture negative^b^	12/71	13/136	0.10
Antimicrobial resistant isolate present	1/71	11/136	**0.04**

^a^Anaerobes included *Clostridium perfringens*, *Bacteroides* species, *Peptostreptococcus* species, *Fusobacterium* species, *Actinomyces* species, *Prevotella* species, *Lactobacillus *species, *Veillonella* species. The prevalence of these organisms was not different between the two cohorts

^b^Patients classified as culture negative had negative blood and aspiration cultures (4/136) or negative blood cultures only when aspiration was not performed (9/136)

### Mortality

In-hospital mortality (as reported by Kaplan et al.), was not different between the cohorts (10/136 or 0.26 per 100,000 population per year from 2015 to 2017 vs 7/71 or 0.22 per 100,000 population per year from 1999 to 2003, p = 0.35). In our study, all-cause 30-day mortality was 7.4% (10/136 or 0.26 per 100,000 population per year). Factors associated with mortality were not outlined by Kaplan et al. In our study, factors associated with 30-day mortality included: polymicrobial bacteremia, no drainage of PLA, history of congestive heart failure or liver disease, and increasing total bilirubin (Table 4). Of subjects who died within 30 days, the mean total bilirubin on admission was 114 (IQR 68–126) vs 27 (IQR 10–29) in those who survived, p < 0.0001.

**Table 4:** Factors associated with 30-day mortality in PLA^a^

Factor	Univariate (OR [95% CI], p-value)	Multivariate (OR [95% CI], p-value)
Polymicrobial bacteremia	17.27 [4.99–59.69], < 0.001	18.5 [1.8–191], 0.014
No drainage performed	5.80 [1.58–21.30], 0.006	13.3 [1.1–167], 0.045
History of congestive heart failure	6.55 [1.85–23.24], 0.04	35.7 [1.4–912], 0.031
History of liver disease^b^	6.06 [1.84–19.96], 0.004	10.3 [0.9–115], 0.059
Total bilirubin	13.0 [1.69–100.94], 0.002^c^	1.0 per umol/L [1.00–1.04], 0.023

^a^30-day mortality data was available for all patients

^b^Includes cirrhosis, hepatitis B, hepatitis C, traumatic liver injury, liver transplantation, hepatolithiasis, hepaticojejunostomy

^c^Univariate analysis conducted for total bilirubin greater than the upper limit of normal

We recorded the use of proton pump inhibitors (PPI) in the community prior to hospitalization and fifty-seven of the 136 subjects (42%) were taking PPIs. When PPI use was analyzed in relation to 30-day mortality, a statistically significant protective effect against mortality was revealed (p = 0.003). None of the 10 patients who died were taking PPIs in the community prior to admission.

## Discussion

With both centralized electronic medical records and comprehensive region-wide laboratory services, the CHZ is an ideal place to study the epidemiology of PLA free of selection bias. The ability to compare our data to that collected 15 years prior using the same data sources allows for consistency that optimizes comparisons in epidemiology over time.

Relative to 1999–2003, we observed an increased incidence rate of PLA in the CHZ. Similarly, Chen et al. noted that the incidence of PLA in Taiwan increased between the years of 2000 and 2011 [1] which was postulated to be the result of a pathogenic clone’s spread [14]. It has since been determined that the hypervirulent mucoviscous *Klebsiella pneumoniae* strains, such as K1 and K2 serotypes, are an important agent of PLA principally in Asia but increasingly in North America [15, 16]. Peirano et al.’s study in the CHZ between 2001 and 2007 demonstrated that 8.2% of bacteremic *Klebsiella pneumoniae* isolates were hypermucoviscous. Of those bacteremic with hypermucoviscous isolates, liver abscess was the most common clinical presentation [17]. The rate of hypermucoviscous *Klebsiella pneumoniae* isolates was not determined in our study. However, the increase in incidence rate in the CHZ in our study is not driven by this—as *Streptococcus anginosus* group remained most prevalent and the proportion of individual pathogens was stable between cohorts. Meddings et al. noted an increase in PLA prevalence in the United States between 1994 and 2005 and hypothesized this was the result of an aging population, increasing prevalence of biliary disease, instrumentation of the biliary tract, diabetes, and liver transplantation. It is possible that the increase in PLA in our cohort could be explained by an increase in prevalence of predisposing comorbidities. The prevalence of liver transplantations in Alberta has increased steadily from 16/100,000 population in 2006 to 24/100,000 population in 2019 [12, 13, 18]. The diabetes prevalence rate in the CHZ has also steadily increased, from 4.88 in 2004 to 7.19 in 2017[10]—and herein we observed an attributable four-fold increased risk of PLA relative to the general population. If these trends continue, we can further expect PLA incidence rate to increase. Additionally, it is possible that increases in instrumentation of the biliary tract in our cohort compared to the 1999–2003 cohort may have also contributed to the rising incidence rate. This was a factor highlighted by Meddings et al. although we were unable to obtain data regarding frequency of biliary procedures between 1999 and 2017 in our region.

Similar to the Kaplan et al. study, most patients in our cohort were microbiologically confirmed as PLA based on liver aspiration culture (65%). This was unchanged between the two cohorts despite fewer aspirations being performed in our cohort. A similar number of diagnoses were confirmed using aspiration because the yield of culture was higher compared to 1999–2003. In terms of diagnosis based on imaging findings, CT scan is a more sensitive modality for detection of PLA, compared to ultrasound [4]. In our cohort, CT scan was a superior imaging modality for the detection of PLA when compared to ultrasound, similar to the 1999–2003 cohort.

PLA was the admitting diagnosis in a greater proportion of subjects in our study compared to the Kaplan cohort. This suggests that over the 15-year time span between the 2 studies, PLA diagnosis has occurred more quickly, possibly secondary to improved access to imaging tests and improved image quality [19].

Biliary sources for PLA remained most common, unchanged from the 1999–2003 cohort. We found an increase in the incidence rate of pylephlebitis and diverticulitis as a cause of liver abscess. One explanation for the higher incidence rate of pylephlebitis is improved access to high resolution imaging studies [20]. This increased observation of pylephlebitis, whether from improved detection or a true increase in incidence, is important because optimal management of these clots is uncertain. The role of anticoagulation in this condition remains controversial. A study of pylephlebitis in Taiwan by Wang et al. showed that PLA is frequently concomitant [21]. They observed *Klebsiella pneumoniae* as the most common pathogen associated with pylephlebitis. Interestingly, none of the patients in our cohort with *Klebsiella pneumoniae* liver abscess had pylephlebitis. The reason for this difference is unknown, but perhaps the difference in serotypes between the two geographic regions plays a role. In our cohort, infections with anaerobic species were significantly associated with diagnosis of pyelophlebitis (p = 0.009).

In our study, liver transplantation, diabetes mellitus, and malignancy were determined to be significant risk factors for the development of PLA, similar to 1999–2003. In contrast, alcohol use disorder was no longer identified as a risk factor. It is possible this was influenced by differing definitions of alcohol use disorder, as the criteria for this in Kaplan et al.’s study was not outlined. In our study, we collected data for “heavy alcohol use” (defined as ≥ 5 alcoholic drinks ≥ 2 times per month for males and ≥ 4 alcoholic drinks ≥ 2 times per month for females) as this was the definition applied to population-level data. Alternatively, this difference may be due to differences in documentation regarding alcohol use between cohorts. Indeed, a Taiwan-based study by Wang et al. concluded that alcohol intoxication is associated with increased risk of PLA [22]. We identified five previously unrecognized population level-risk factors for PLA (ischemic heart disease, chronic obstructive pulmonary disease, cirrhosis, history of congestive heart failure, and end-stage renal disease). Although many studies list clinical characteristics seen in their patients with PLA, few compare this to the prevalence among the general population which is a unique feature of the CHZ data—and key to understand risk factors.

The pathogens implicated in PLA in our cohort were not significantly different than those observed in the 1999–2003 cohort and *Streptococcus anginosus* group continue to be most prevalent, consistent with other North America studies [2]. Continued prospective surveillance for hypermucoviscous -*K. pneumoniae* are key in monitoring for a shift in epidemiologic trends in this North American population. Importantly, increasing prevalence in hypervirulent *Klebsiella pneumoniae* stains could impact patient management as they are more likely to be monomicrobial and result in metastatic spread.

Globally, antimicrobial resistance is a concern in a variety of infections, including PLA [23]. With increasing prevalence of ESBL-producing and carbapenem-resistant organisms on the rise in Canada [24, 25], identification of the causative organisms in PLA and determination of susceptibility profiles is increasingly important to ensure culture-optimized antimicrobial therapy. In our study, the rate of antimicrobial resistant isolates compared to 15 years ago was increased—highlighting the importance of achieving a definitive microbiologic diagnosis [26]. The declining frequency of liver aspirations in the CHZ compared to approximately 15 years ago is something that may benefit from quality improvement interventions.

It is disappointing that despite innovation and advances in care, mortality has not changed significantly compared to 1999–2003. This highlights the need for further study into management and treatment strategies as practices are quite variable. It has been established previously that abscess drainage is important in management [27] and the fact that “no abscess drainage” was associated with 30-day mortality in our study provides further support for this notion. The development of clinical practice guidelines for PLA in North America will be an important advance in the field.

An interesting observation in this study was the potential protective effect of PPI-use against PLA-mortality. This contrasts a study by Bettinger et al. that investigated the effect of PPI treatment on mortality in patients with PLA. They found that PPI-treatment was associated with significantly higher index mortality and 90-day mortality [28]. In their study, the multivariate analysis examining PPI treatment included PPI-use in the community prior to admission as well as use during their hospitalization. Our study differs as we only analyzed PPI use in the community prior to hospitalization. This raises questions around whether there are differing impacts on mortality in PLA if PPIs are used in the community prior to admission versus in hospital during acute illness. Another study by Lin et al. investigated the use of PPIs and risk of development of PLA and concluded that PPI use conferred a 7.59-fold increased risk of PLA [29]. In our study, 42% of subjects were taking a PPI prior to hospitalization but population-level data was not available to determine if PPI use was associated with increased PLA risk. PPIs are commonly prescribed medications [29] and their impact on the development of PLA and associated-mortality is an important area that warrants further investigation.

Multiple limitations are important to note in our study. We used complementary strategies relying on clinical and microbiologic diagnosis to increase our capacity to identify all PLA CHZ cases, however, cases that were misdiagnosed or not recognized as PLA would have been missed. Due to differing infrastructure in the medical system responsible for Pediatric care, children were not included in this study. Additionally, because of the way census data is divided, the age cutoff of 20 years (as opposed to 18) was utilized. Determination of comorbidities and PLA source was determined by reviewing patient charts, including investigations, discharge summaries, and consultation notes generated by physicians. Because of this, the accuracy of the data set is dependent on the accuracy of the investigations and documentation. Although a rigorous population-based design was utilized, there are limitations to our population-based assessment [30]. We are unable to assess independent risk factors for acquisition of PLA using logistic regression. This is because we estimated the rates of underlying illnesses in the population-at-large based on current disease prevalence registries, but do not have individual linked data on all CHZ residents. This limitation is nearly universal to population-based study designs [31, 32]. There are also inherent limitations in using ICD-9 codes for defining co-morbid illness. Lastly, our data sets were limited in terms of the availability of some data. Important factors, such as differences in the use of certain medications (e.g. immunosuppressive agents and oral hypoglycemic agents), were not available which may have contributed to some of the differences between the two cohorts.

## Conclusions

PLA in the CHZ has increased in incidence over the last 15 years. The increasing prevalence of comorbidities in our population and factors that are associated with risk of PLA suggests this will continue to be an emerging diagnosis of concern. Increasing antimicrobial resistance highlights the importance of future studies to generate an evidence base for the optimal antibiotic treatment for this condition.

## Data Availability

The datasets generated during the current study are not publicly available due to patient confidentiality but are available from the corresponding author on reasonable request. Four additional datasets were analyzed during the current study: (1) Government of Alberta. Interactive Health Data Application [Internet]. [cited 2020 Jun 11]. Available from: http://www.ahw.gov.ab.ca/IHDA_Retrieval/selectCategory.do?dataBean.id=6&command=doSelectSubCategory&cid=6. (2) Statistics Canada. Census Profile, 2016 Census [Internet]. Calgary, AB; 2017. Available from: https://www12.statcan.gc.ca/census-recensement/2016/dp-pd/prof/details/page.cfm?Lang=E&Geo1=CSD&Code1=4806016&Geo2=PR&Code2=01&Data=Count&SearchText=4806016&SearchType=Begins&SearchPR=01&B1=All&Custom=&TABID=3. (3) Canadian Institute for Health Information. Treatment of End-Stage Organ Failure in Canada, Canadian Organ Replacement Register, 2006 to 2015: Data Tables, Liver Transplants. Ottawa, ON; 2016. Available from: https://www.cihi.ca/sites/default/files/document/liver_transplant_section_v0.1_en_2017.xlsx. (4) Canadian Institute for Health Information. Treatment of End-Stage Organ Failure in Canada, Canadian Organ Replacement Register, 2010 to 2019: Extra-Renal Transplants — Data Tables. Ottawa, ON; 2020. Available from: https://www.cihi.ca/sites/default/files/document/extra-renal-transplants-2010-2019-data-tables-en.xlsx.

## Abbreviations

PLA: Pyogenic liver abscess
CHZ: Calgary Health Zone
ESBL: Extended spectrum beta-lactamase
ICD: International Classification of Diseases
CLS: Calgary Laboratory Services
MRSA: Methicillin-resistant *Staphylococcus aureus*
VRE: Vancomycin-resistant *Enterococcus*
IQR: Interquartile ranges
CI: Confidence interval
OR: Odds ratio
CT: Computed tomography
PPI: Proton pump inhibitor
